# Supplementary material for: Cell morphology and nucleoid dynamics in dividing Deinococcus radiodurans
Source: Nat Commun. 2019 Aug 23;10:3815. doi: 10.1038/s41467-019-11725-5 (PMC6707255; doi:10.1038/s41467-019-11725-5)
Supplement: Supplementary file 1 — Supplementary Information [file 41467_2019_11725_MOESM1_ESM.pdf]

Cell morphology and nucleoid dynamics in dividing *Deinococcus radiodurans*

Floc'h et al.

Supplementary Information

Supplementary Figures 1-9

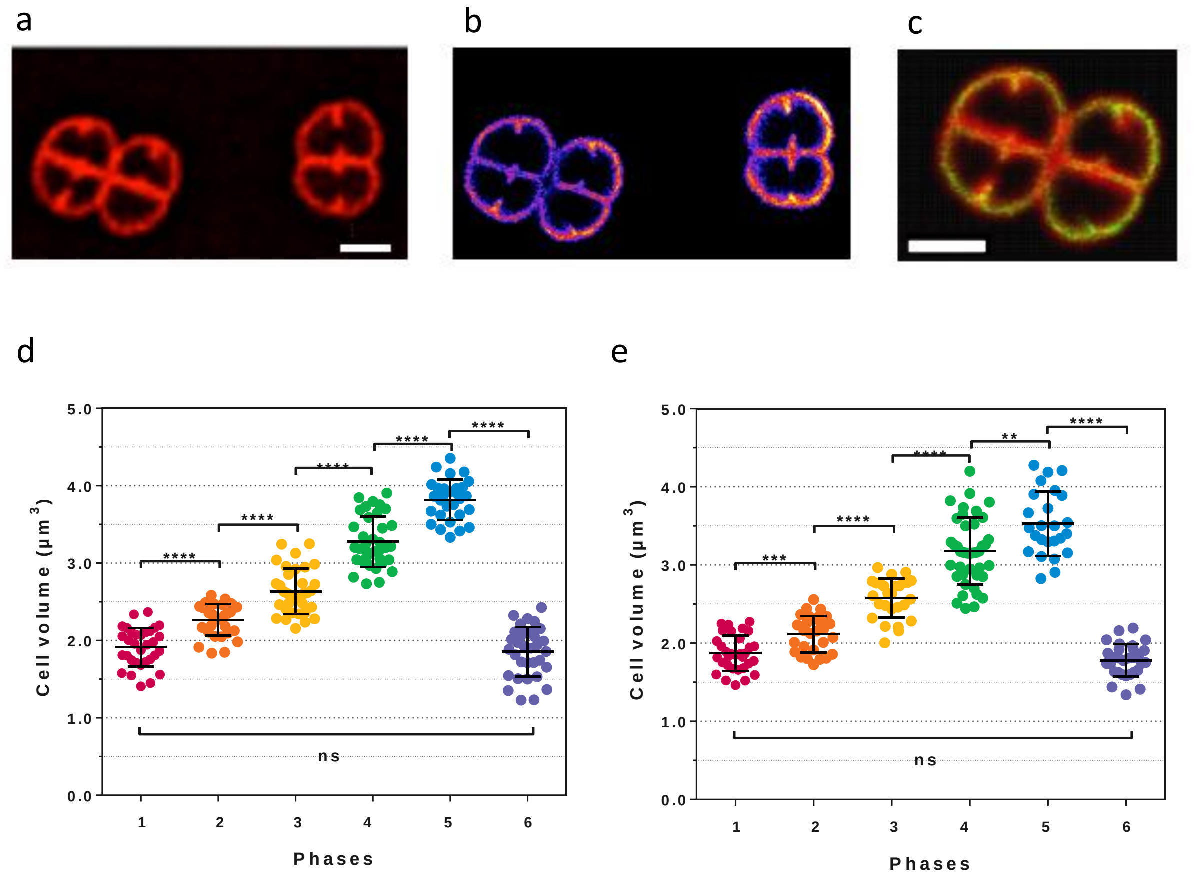

Supplementary Figure 1: Comparison of spinning-disk and PAINT images of *D. radiodurans* cells stained with the membrane dye Nile Red. (a) Spinning-disk image and (b) PAINT image of the same Nile Red stained *D. radiodurans* cells. (c) Overlay of images (a) and (b). Scale bar: 1 µm. (d)-(e) Changes in cell volume as a function of the cell cycle retrieved from either spinning-disk confocal images (d) or super-resolved, PAINT images (e) of Nile Red stained *D. radiodurans*. The same three independent populations of cells were observed with the two imaging techniques. (N=180 cells, N>25 for each phase). Data are represented as mean ± SD. Individual values are shown as dots. Cell volumes were calculated by measuring the cell parameters as described in Fig. S2. Only very small differences in cell volumes were observed between the two imaging methods. ***: P<0.001, ****: P<0.0001, ns: not significant; statistical test: non-parametric Mann-Whitney. Source data are provided as a Source Data file

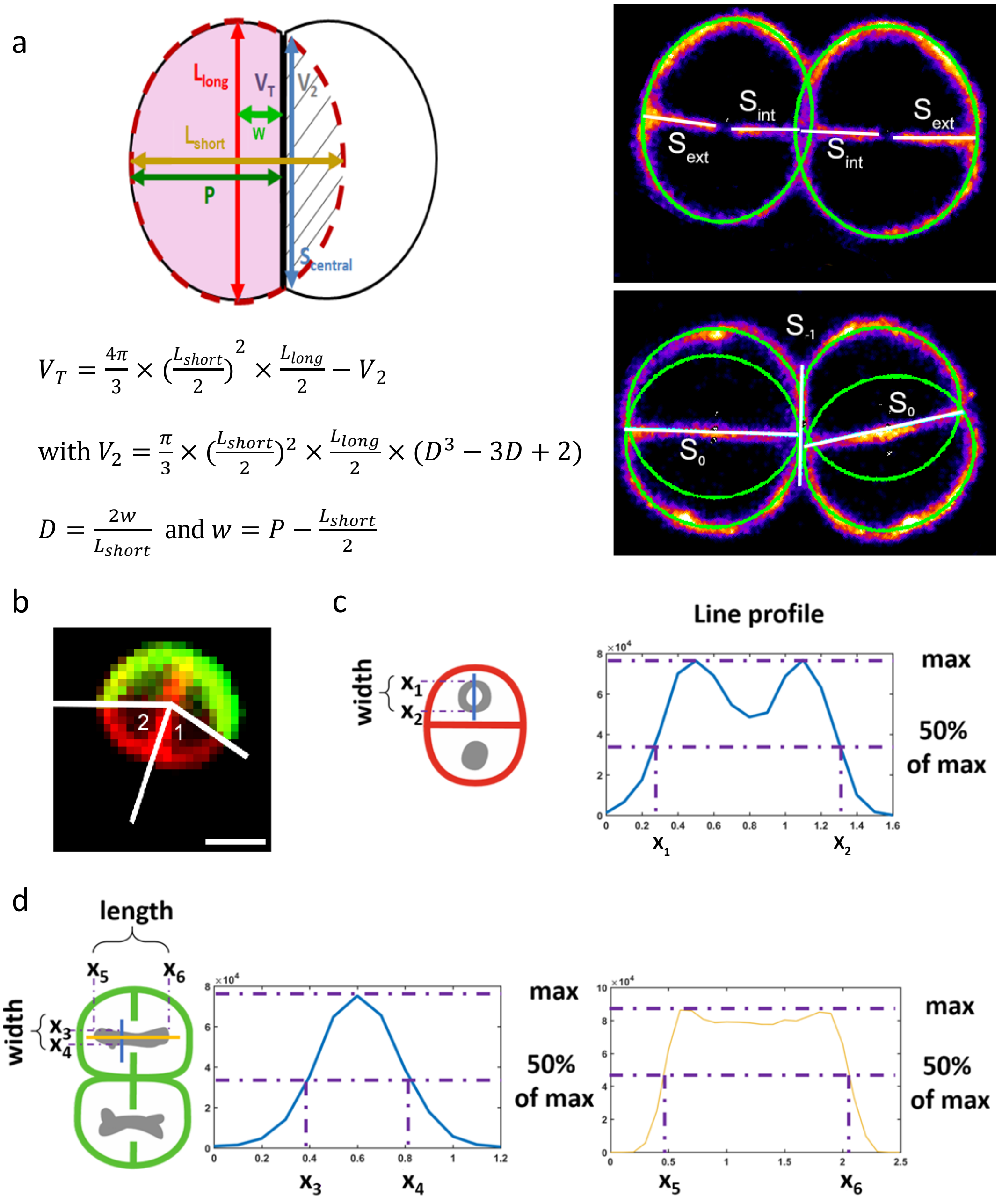

Supplementary Figure 2: Cell and nucleoid measurements used in this study. (a) Schematic diagram illustrating the mode of calculation of the volumes of *D. radiodurans* cells based on the fitting of ellipses (dotted red line) to individual cells and the equations used for these calculations. L_{long}= length of major axis of the fitted ellipse; L_{short}= length of minor axis of the fitted ellipse; P= distance between central septum and the opposite side of the ellipse; w= distance from center of ellipse to central septum (=P-[L_{short}/2]); S_{ext}=length of exterior septum of cells in Phases 4 and 5; S_{int}= length of interior septum of cells in Phases 4 and 5; S_0= length of the newly formed septum in Phase 6; S_{-1} = length of the septum originating from the previous cell cycle. (b) Illustration of the two angle measurements made to determine the lengths of the BADA (green) and Nile Red (red) labelled cell perimeters in Phase 1 diads at T= 130 min. Scale bar: 1 µm. (c)-(d) Illustration of the measurements made to determine the width of the toroidal-shaped nucleoids (c) and the length and width of the elongated nucleoids (d).

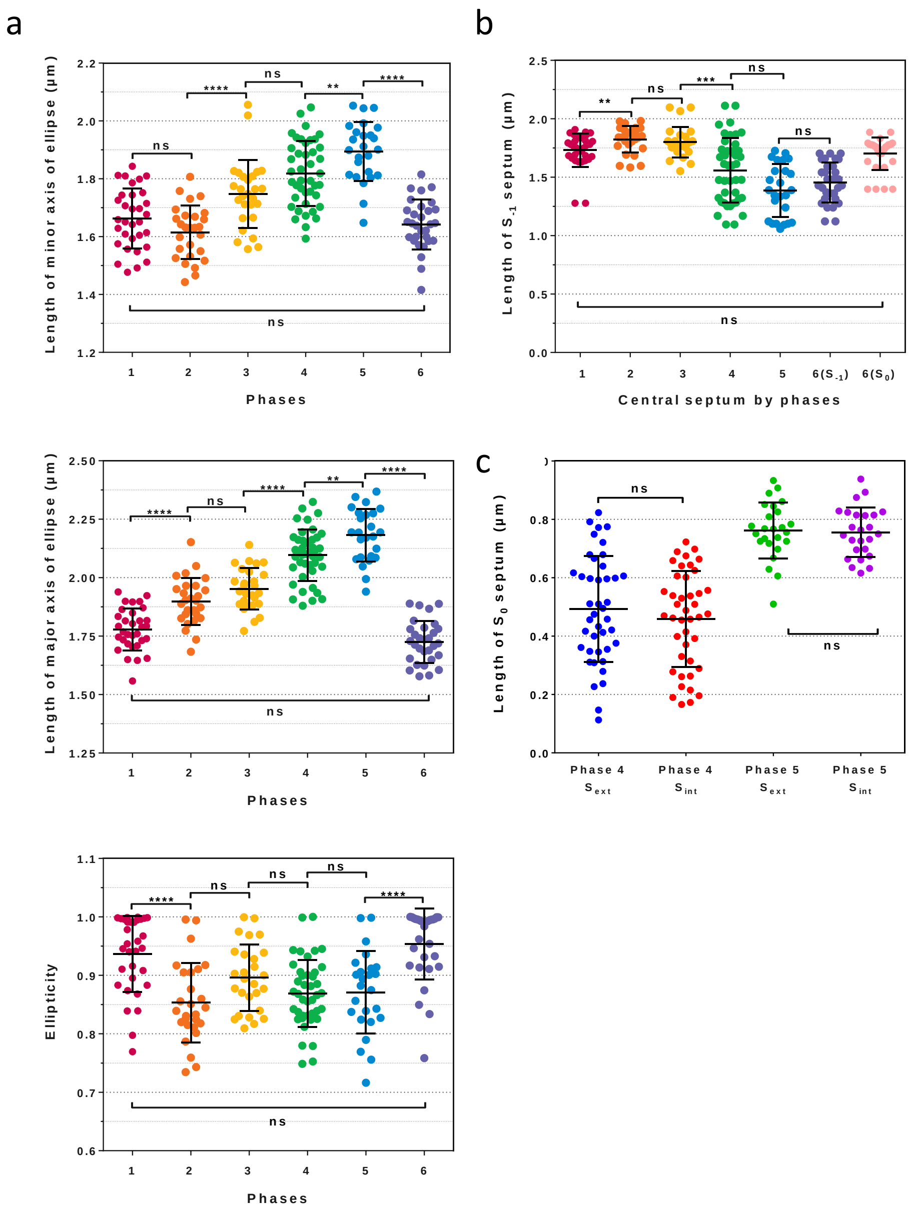

Supplementary Figure 3: Cell parameters of *D. radiodurans* cells extracted from PAINT images of Nile Red stained, exponentially growing cells. (a) Lengths of the minor (top) and major (middle) axes of the fitted ellipses used to measure cell volumes (see Supplementary Figure 2), and ellipticity (ratio between the length of the minor and major axes) of the cells (bottom) as a function of the cell cycle phases. (b) Lengths of the septa originating from the previous cell division (S_{-1}; top) as a function of the cell cycle phases. The decrease in the length of S_{-1} illustrates the increased invagination at the junction between the two cells forming a diad. For tetrads in Phase 6, the lengths of both the S_{-1} and S_0 septa are shown for comparison. (c) Lengths of the growing exterior and interior S_0 septa in Phases 4 and 5 (see Supplementary Figure 2). N=180 cells, N>25 for each phase. Data are represented as mean ± SD. Individual values are shown as dots. *: P<0.05, **: P<0.01, ***: P<0.001, ****: P<0.0001, ns: not significant; statistical test: non-parametric Mann-Whitney. Source data are provided as a Source Data file.

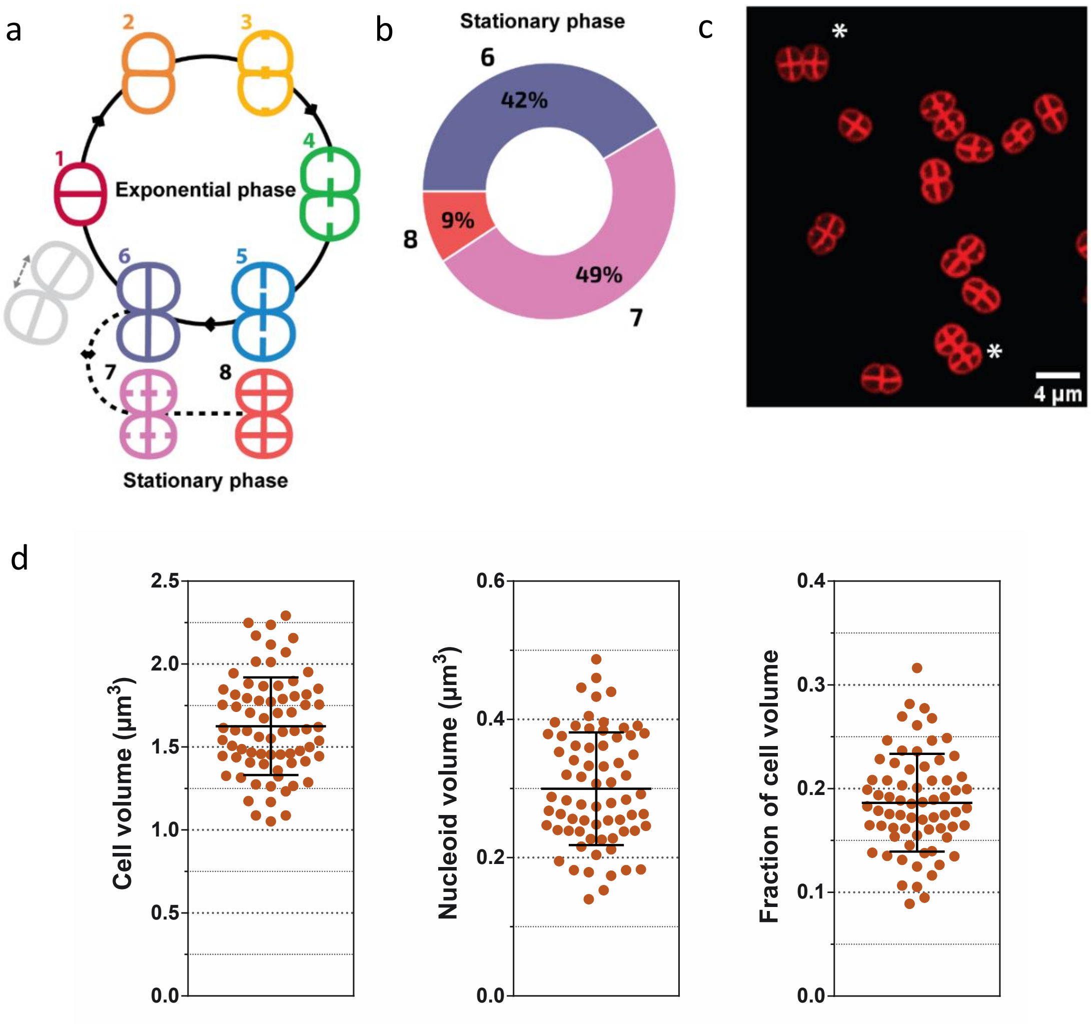

Supplementary Figure 4: Changes in cell morphology during the stationary phase of *D. radiodurans* cell growth. (a) Schematic representation of the cell cycle, for one diad. In stationary phase, two additional phases can be seen: Phase 7 corresponds to tetrads engaging in a new cell cycle without dissociation into diads, and Phase 8 cells are octads. (b) Distribution of phases in the population of stationary cells (24h of growth) when observed at a given time point (N>600). All data were collected from at least 2 independent experiments. (c) Spinning-disk image of Nile Red stained *D. radiodurans* stationary cells. Octad cells are marked with an asterisk. Scale bar: 4 µm (d) Mean cell and nucleoid volumes, and fraction of the cell volume occupied by the nucleoid in stationary phase 6 tetrads (N=69 cells). Cell volumes were calculated by measuring the cell parameters presented in Supplementary Figure 2 on spinning-disk images. Nucleoid volumes were measured as described for exponentially growing *D. radiodurans* cells (see Methods). Data are represented as mean ± SD. Individual values are shown as dots. Source data are provided as a Source Data file.

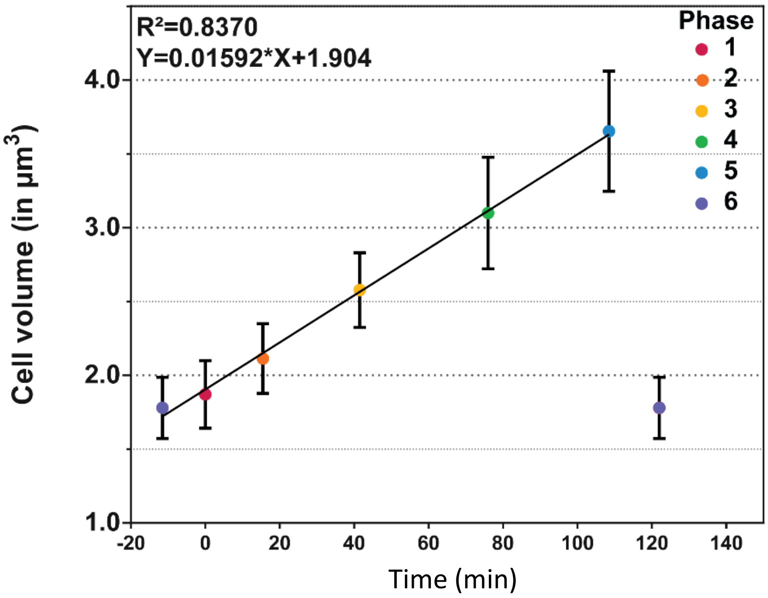

Supplementary Figure 5: Increase in *D. radiodurans* cell size as a function of time. Linear increase of *D. radiodurans* cell volume with time during the cell cycle, derived from measurements of Nile Red stained exponentially growing cells imaged with PAINT (see Fig. 1d). The start of Phase 1 was defined as time 0 and the subsequent data points correspond to the start of the subsequent phases (2-6) based on the mean durations of the phases determined in timelapse experiments (Fig. 1e). The data points corresponding to mean values ± SD were fitted to a linear regression (R^2 value for the fit and equation of regression are given in top left).

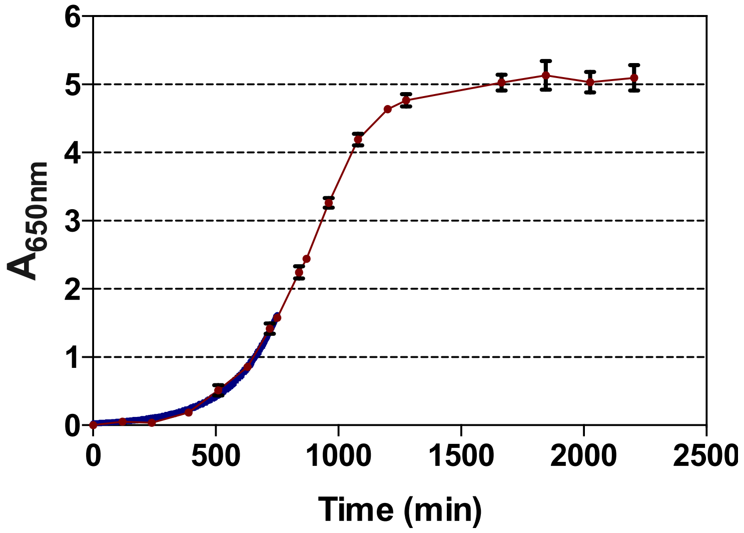

Supplementary Figure 6: Growth curve of wild-type *D. radiodurans* cells. Growth curve of *D. radiodurans* cells grown in TGY2X medium at 30°C in a shaking incubator established using absorbance at 650nm (A_{650nm}) measurements from at least three independent cultures. The doubling time of exponentially growing cells was derived by fitting the initial growth phase to an exponential growth curve (blue; see Methods) and was estimated to be 130 minutes. Source data are provided as a Source Data file.

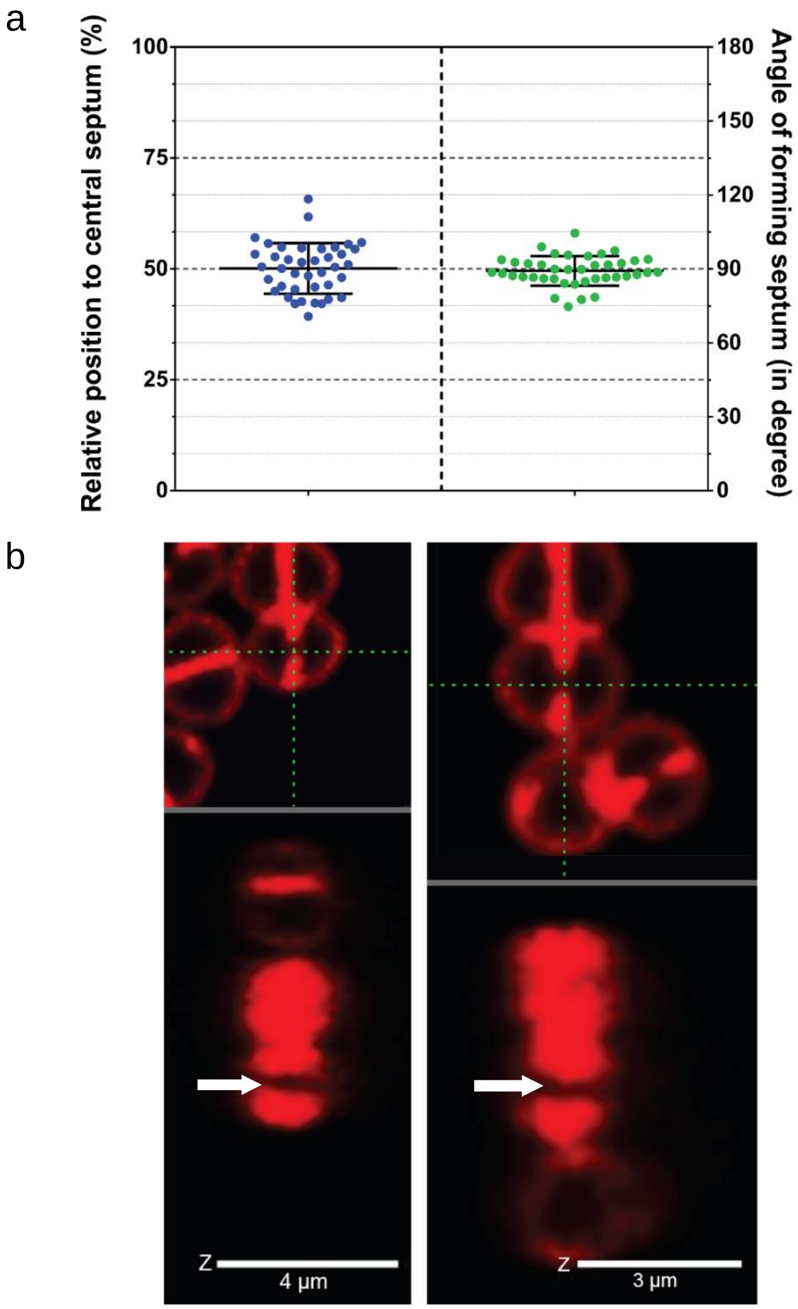

Supplementary Figure 7: Septal growth and closure mechanism in *D. radiodurans*. (a) Position and angle of newly forming septa relative to the central septum (N>40 cells)**.** Data are represented as mean ± SD. Individual values are shown as dots. (b) Two examples (left and right) of 3D spinning-disk images of Nile Red stained *D. radiodurans* cells in which septal closure is in progress. The top panel represents the observed cells, in the XY plane, with the observation cross superimposed in green (dashed line). The bottom panel is the XZ orthogonal view along the X direction defined by the green cross in the top panel. Septal growth from both sides of the cells leaves a gap stretching all away across the height of the cell, as seen in the XZ projection and indicated with white arrows. These images support a closing door mechanism rather than a diaphragm. Source data are provided as a Source Data file.

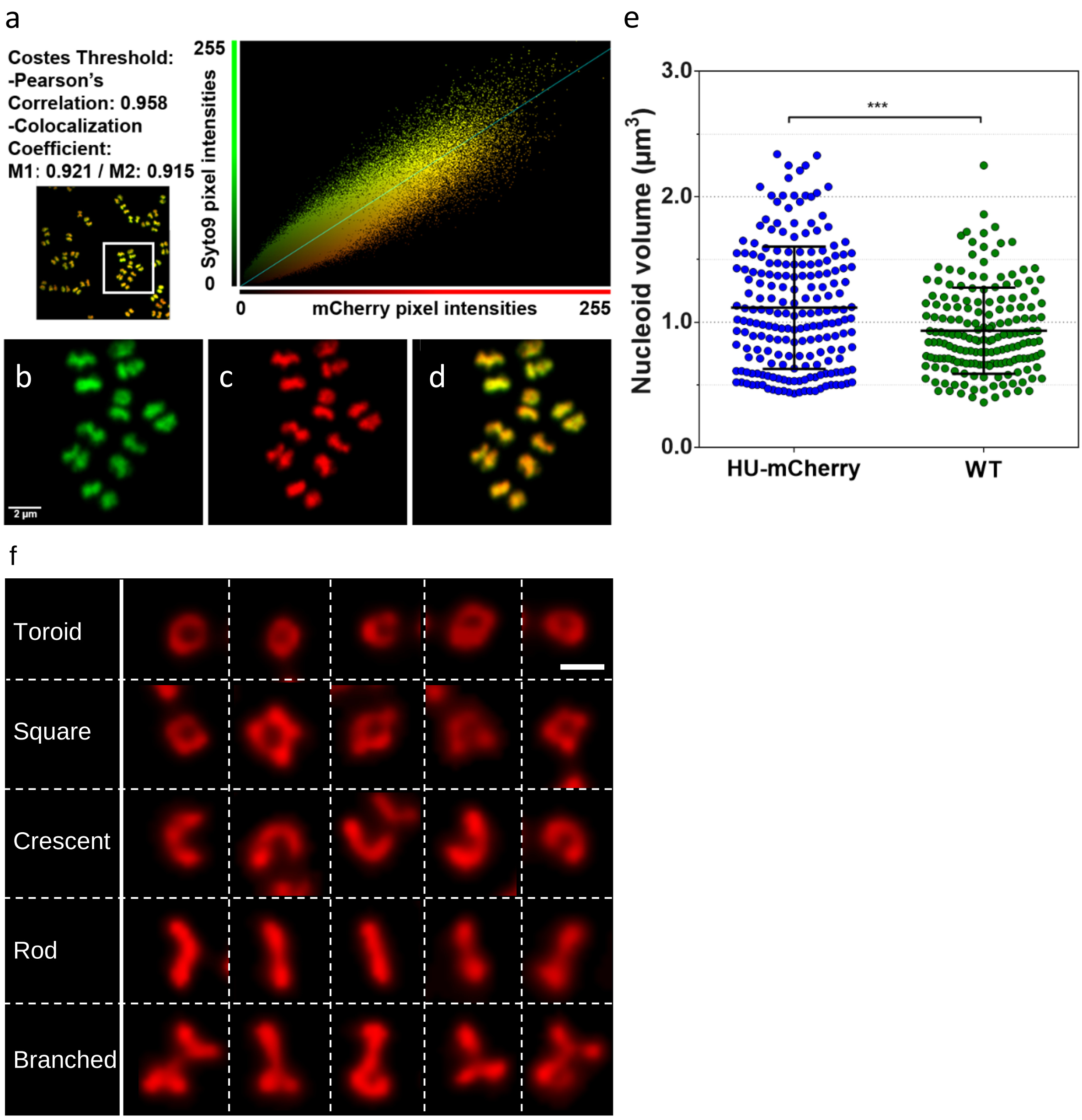

Supplementary Figure 8: Nucleoid labelling in exponentially growing *D. radiodurans.* (a) Colocalization statistics (analysis with Volocity software) of HU-mCherry and Syto9 fluorescence signals in *D. radiodurans* cells expressing HU-mCherry and stained with Syto9. The colocalization analysis was performed on the full Z-stack of untreated raw images. (b)-(d) Close-up insets of white box in (a), showing nucleoids stained with both Syto9 (b) and HU-mCherry (c). (d) Overlay of the two fluorescence signals. The images correspond to raw images, shown in extended focus (all Z-planes are superimposed into a single, final image). (e) Nucleoid volumes of Syto9 stained exponentially growing wild-type (WT) and HU-mCherry expressing *D. radiodurans* cells (N>175; ***: P<0.001; statistical test: non-parametric Mann-Whitney). Data are represented as mean ± SD. Individual values are shown as dots. (f) Array of the representative shapes of *D. radiodurans* nucleoids seen in HU-mCherry labelled *D. radiodurans* cells (extracted from timelapse presented in Supplementary Movie 3). Scale: 1 µm. Source data are provided as a Source Data file.

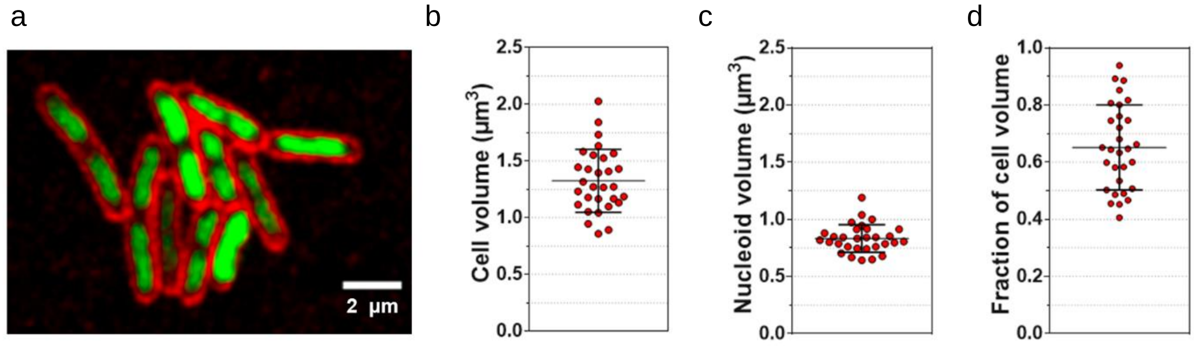

Supplementary Figure 9: Nucleoid labelling in exponentially growing *E. coli* BL21 cells. (a) Image of exponentially growing *E. coli* BL21 cells stained with Syto9 and Nile Red. (b) Cell volume, (c) nucleoid volume and (d) fraction of the cell volume occupied by the nucleoid (N>30) in *E. coli* cells. The fraction consists of the ratio of the nucleoid volume divided by the volume of the associated cell. The cell volume was computed from measurements of the length and width of individual cells, assuming *E. coli* cells were cylinders capped with two semi-spheres. The nucleoid volumes were extracted as for *D. radiodurans* nucleoids (see Methods). Data are represented as mean ± SD. Individual values are shown as dots. Source data are provided as a Source Data file.
