# Supplementary material for: Cell morphology and nucleoid dynamics in dividing Deinococcus radiodurans
Source: Nat Commun. 2019 Aug 23;10:3815. doi: 10.1038/s41467-019-11725-5 (PMC6707255; doi:10.1038/s41467-019-11725-5)
Supplement: Supplementary file 3 — Description of Additional Supplementary Files [file 41467_2019_11725_MOESM3_ESM.pdf]

## Description of Additional Supplementary Files

File Name: Supplementary Movie 1
Description: **Growth and division of *D. radiodurans.*** Time-lapse movie of live, Nile Red stained, exponentially growing *D. radiodurans* cells, deposited on an TGY2X agarose pad. Images were acquired every 10 min for a total period of 4 h. Scale bar: 2 µm.

File Name: Supplementary Movie 2
Description: **Nucleoid dynamics in live, wild-type *D. radiodurans*.** Time-lapse movie of live, Syto9 stained, *D. radiodurans* cells, deposited on an TGY2X agarose pad. Images were acquired every 10 min for a total period of 3 h. Scale bar: 5 µm.

File Name: Supplementary Movie 3
Description: **Nucleoid dynamics in live, HU-mCherry expressing *D. radiodurans*.** Time-lapse movie of live, HU-mCherry expressing *D. radiodurans* cells, deposited on an TGY2X agarose pad. Images were acquired every 5 min for a total period of 3 h. Scale bar: 5 µm.

File Name: Supplementary Movie 4
Description: **Simulation of the major morphological changes occurring at the cellular and nucleoid level in dividing *D. radiodurans.*** On-scale (size and timing) simulation of *D. radiodurans* cell cycle, illustrating the coordination of the morphological changes of the nucleoids with septal growth during the cell cycle. This movie was created based on the cell and nucleoid measurements presented in Fig. 1d, Fig. 4a and Supplementary Figure 3 and on the cell cycle duration presented in Fig. 1e. Scale bar: 1 µm.

File Name: Supplementary Movie 5
Description: **Minute-scale dynamics of *D. radiodurans* nucleoids.** Time-lapse movie of live, HU-mCherry expressing *D. radiodurans* cells, deposited on an TGY2X agarose pad. Images were acquired every 20 sec for a total period of 10 min. Scale bar: 4.2 µm.
